# Analysis of Gut Microbiota in Patients with Coronary Artery Disease and Hypertension

**DOI:** 10.1155/2021/7195082

**Published:** 2021-12-27

**Authors:** Chuanqi Wan, Chen Zhu, Gulei Jin, Min Zhu, Junyi Hua, Yuzhou He

**Affiliations:** ^1^The First Affiliated Hospital of Zhejiang Chinese Medical University, Hangzhou, China; ^2^Guhe Information Technology Company, Hangzhou, China

## Abstract

Cardiovascular and cerebrovascular diseases are characterized by high rates of morbidity and mortality. Microbiota is closely associated with cardiovascular disease. We aimed to comprehensively analyze the microbiotas of 300 healthy controls, 300 patients with high blood pressure (HBP), and 300 patients with coronary heart disease (CHD). The results indicated no significant difference in microbiota diversity among the three groups (*P* > 0.05). However, differences in microbiota richness among the three groups were significant (*P* < 0.05). Bacteroidetes and Bacteroidia were the dominant bacteria in the CHD group, Enterobacteriales and *Escherichia-shigella* in the HBP group, and Acidaminococcaceae and *Phascolarctobacterium* in the healthy control group. The prediction results of the random forest model indicated that the population with CHD displayed prominent features with high sensitivity, indicating that microbiota detection might become a novel clinical indicator to predict and monitor the risk of cardiovascular events. The prediction of microbiota function suggested differences in oxygen supply and chronic inflammation between populations with HBP/CHD and healthy populations. Although there is no difference in gut microbiota diversity among the three groups, each group has its dominant microbiota in terms of richness.

## 1. Introduction

Human gut microbiota is believed to be directly or indirectly involved in cardio-cerebrovascular disease (CVD) [1]. Although cause-effect relationships have not been established, the reported associations between gut microbiota and CVD alterations are prominent [2]. Studies [3–7] have shown that gut microbiota is associated with obesity, diabetes, dyslipidemia, and hypertension, which are risk factors for coronary heart disease. Gut microbiota can induce atherosclerosis and coronary heart disease through metabolites involved in cholesterol metabolism, uric acid metabolism, oxidative stress, and inflammation [8–10].

For example, microbiota produced trimethylamine (TMA) through the metabolism of dietary choline, phosphatidylcholine, and L-carnitine, which was further metabolized to a proatherogenic species, trimethylamine-N-oxide (TMAO), thus promoting the occurrence of atherosclerosis (AS) [11–13]. Santisteban et al. [14] suggest that patients with high blood pressure (HBP) are associated with obvious gut microbiota disturbance and intestinal mucosal barrier dysfunction. Li et al. [15] adopted metagenomic and metabolomic approaches to analyze the gut microbiota characteristics in healthy controls, patients with prehypertension, and patients with primary HBP. The results showed that two patient groups exhibited significantly reduced microbial abundance and diversity. In addition, probiotics reduced the excessive growth of *Prevotella and Klebsiella.* Kim et al. [16] investigated the fecal samples from 22 patients with HBP and eighteen healthy controls with normal blood pressure. Their results suggested that the fecal flora composition and function in patients with HBP significantly changed, and the fecal content of butyrate significantly decreased. In terms of function prediction, it was discovered that the abundance of Streptococcus positively correlated with blood pressure, and that of *Enterobacteriaceae* positively correlated with the myocardial index.

Researchers have paid increasing attention to the relationship between gut microbiota and CVD. However, the limited clinical sample size may lack universality and persuasiveness of previous studies. Large-sample studies on the 16S RNA-based high-throughput sequencing of gut microbiota in HBP and CHD are deficient. Consequently, more clinical studies are needed to obtain more details about the changes in intestinal microbial compositions and their effects on HBP and CHD [17].

In this study, fecal microbiota from 300 healthy controls, 300 patients with HBP, and 300 patients with CHD was profiled by 16S ribosomal DNA. Metagenomic sequencing was performed on 900 samples. Bacterial community structures and metabolic function between the samples were assessed. Specifically, we aimed to explore the differences of gut microbiota among these populations to provide further a theoretical foundation for preventing HBP and CHD.

## 2. Materials and Methods

### 2.1. Objects

A total of 300 patients with HBP and 300 patients with CHD who were admitted to the First Affiliated Hospital of Zhejiang Chinese Medical University between 2017 and 2019 were enrolled in the present study. HBP was defined as blood pressure higher than 140/90 mmHg, and patients in the CHD group were confirmed to have coronary artery disease by coronary angiography. At the same time, 300 healthy volunteers were selected from the Physical Examination Center of our hospital as controls.

The criteria included participants who (1) did not take antacids, probiotics, antibiotics, or antibacterial agents within 30 days of the sample collection; (2) were aged 18–80 years old; (3) had a BMI of 18–35 kg/m^2^; (4) had no organic disease in the digestive system; (5) had not undergone gastrointestinal surgery; (6) had no history of diseases that might affect the gut microbiota, such as alcoholism, diabetes, tumor, heart failure, kidney failure, or stroke; (7) did not take high blood pressure drugs or coronary heart disease drugs; and (8) had signed informed consent documents. The characteristics of the three groups are listed in Table 1. The ethics committee of the First Affiliated Hospital of Zhejiang Chinese Medical University approved all study protocols (number: 2017-KL-041-01). Informed consent was obtained after written introductions were provided to all 900 volunteers from the Hangzhou in the Zhejiang Province of China.

### 2.2. Sample Collection and DNA Extraction

The fecal samples of patients were collected using a sterile swab into the sampling tube containing the preservation fluid. Afterward, DNA was extracted using the sodium dodecyl sulfonate (SDS) lysate freezing-thawing method. The genomic DNA was extracted using a PowerMax extraction kit (MoBio Laboratories, CA, USA) and preserved at −20°C. Then, the DNA quantity and quality were determined using a NanoDrop ND-1000 spectrophotometer (Thermo Fisher Scientific, MA, USA). Subsequently, agarose gel electrophoresis was performed. Incidentally, this extraction method has also obtained a Chinese national invention patent (ZL201511009389.7).

### 2.3. 16S RNA-Based Amplicon Pyrosequencing

The polymerase chain reaction (PCR) forward amplification primer in the V4 region of the bacterial 16S rRNA gene was 515F (5′-GTGCCAGCMGCCGCGGTAA-3′), and the reverse primer was 806R (5′-GGACTACHVGGGTWTCTAAT-3′). Later, the barcode was synthesized into the sequence by adopting the specific 7-bp sequence. The PCR reaction system was 50 *μ*L, including 25 *μ*L of high-fidelity enzyme Phusion High-Fidelity PCR Master Mix with HF buffer, 3 *μ*L (10 *μ*M) of respective forward and reversed primers, 10 *μ*L of DNA template, 3 *μ*L of DMSO, and 6 *μ*L of ddH2O. We then used the prepared PCR system for PCR amplification under the following reaction conditions: initial denaturation at 98°C for 30 s, followed by denaturation at 98°C for 15 s, annealing at 58°C for 15 s, and extension at 72°C for 15 s for 25 cycles, and the final extension at 72°C for 1 min. The PCR products were then purified using AMPure XP Beads (Beckman Coulter, IN, USA) and quantified using a PicoGreen dsDNA Assay Kit (Invitrogen, CA, USA). After quantification, an Illumina Novaseq 6000 pair-end 2 × 150 bp platform was adopted for sequencing.

### 2.4. Sequence and Statistical Analyses

Data from each sample were divided from the raw data according to the barcode sequence and the primer sequence. After removing the barcode and primer sequences, the reads of each sample were spliced using the VSEARCH software (v2.4.4, 2017) to obtain raw data. At the same time, the sequence quality was controlled and filtered. The criteria for filtering the low-quality sequences were as follows: sequences of <140 bp, those with the average quality value of <20 bp, those containing unclear basic groups, and those containing single nucleotide repeats of >8 bp. The chimera sequences were eliminated to obtain ultimately useful data.

The operational taxonomic unit (OTU) was analyzed using the VSEARCH software (v2.4.4, 2017), including the removal of repeated sequences and elimination of chimera. Sequences with 97% similarity were clustered into OTUs by default. The representative sequences of OTUs were selected using the default parameters. The representative sequences were conducted using species annotations based on the SILVA database (v128, 2017) using VSEARCH to predefine the OTU list further. Afterward, we determined the intestinal flora composition of each sample at diverse classification levels, including kingdom, phylum, class, order, family, genus, and species. Moreover, the abundance and classification of all OTUs in each sample were recorded, and OTUs <0.001% complete sequences were removed from all samples.

We applied the R package (v3.2.0, 2015.04) to analyze the distribution of sequence length in all samples. We used OTU tables to record the abundance of each OTU of samples. We calculated taxon abundance at the levels of phylum, class, order, family, genus, and species and statistically compared the abundance among groups by Kruskal test from R stats package (metagenomeSeq packages). Based on the OTU table in the Quantitative Insights into Microbial Ecology (QIIME2, 2020.06), alpha diversities including Chao1, Simpson, and Shannon were calculated. The significant differences of alpha diversity metrics were performed using the *R* package “Vegan.” To investigate the structural variation of microbial communities, we performed and visualized beta diversity analysis using UniFrac distance metrics via principal component analysis (PCA), principal coordinate analysis (PCoA), and nonmetric multidimensional scaling (NMDS) from QIIME. We used the R package “vegan” and PERMANOVA (permutational multivariate analysis of variance) to evaluate differences in microbiota among groups. We conducted the linear discriminant analysis (LDA) effect size (LEfSe) method based on the Kruskal–Wallis test and linear discriminant analysis (LDA) to identify significant differentially abundant taxonomy between different groups. We performed random-forest classification for discriminating the samples from different groups using the R package “random forest,” with 1,000 trees and all default settings, and we then used the “pROC” package for receiver operating curve (ROC) analysis. The expected “baseline” error was also included, which was obtained by a classifier that simply predicts the most common category label. Based on 16S rRNA marker gene sequences, we predicted a microbial function using the BugBase (v.0.1.0, 2017).

## 3. Results and Discussion

### 3.1. Results

The sequence statistics in Table 2 are based on the intestinal flora genomes of 900 subjects sequenced by a Novaseq high-throughput sequencer. For example, the total number of sequences in the healthy control group was 12,403,888, the average number of sequences sample was 41,346, the maximum number of sequences was 60,761, the minimum number was 28,043, and the OTUs numbered 628.

#### 3.1.1. Alpha-Diversity Analysis

**Figure 1:**
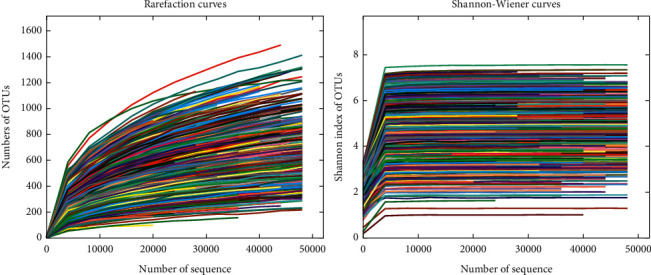
The rarefaction curve of OTUs.

As shown in Figure 1, the rarefaction curve of OTUs tends to be flat, but with the increase of sequencing depth, there will be new OTUs. However, in the Shannon–Wiener curve, which can represent the diversity of intestinal flora; when the maximum sequencing amount is reached, the curve is completely stable, indicating that the bacterial diversity of samples in this study has been completely detected. With the increase of sequencing depth, there may be new species, but the discovery of these new species may have little impact on the study of intestinal flora diversity.

**Figure 2:**
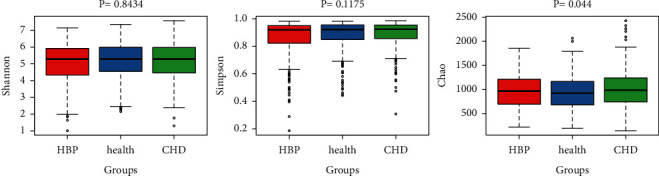
Species richness and species diversity.

Samples in the HBP, CHD, and healthy control groups were statistically analyzed by Kruskal–Wallis test and correlation analyses. The results suggested that the richness levels in samples from the HBP and CHD groups were significantly higher than those in the healthy control group (*P*=0.044), but with no significant difference in the diversity levels (*P*=0.8434 and *P*=0.1175) (Figure 2).

#### 3.1.2. Beta-Diversity Analysis

**Figure 3:**
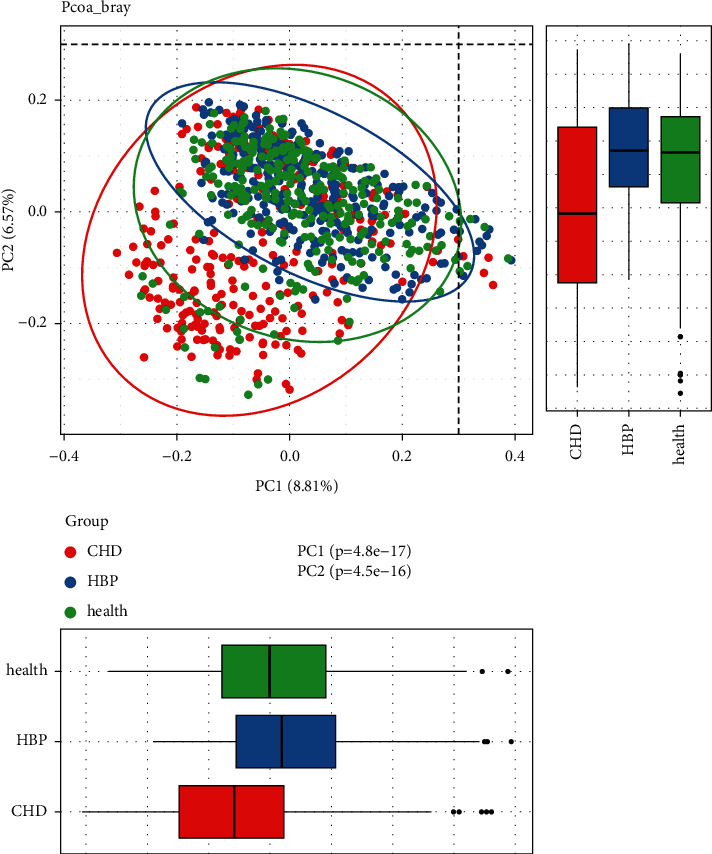
PCoA analysis (the analysis indicated the different community composition in OUT-level among the three groups, with variances of PC1 8.81% and PC2 6.57%).

We used the weighted UniFrac distance algorithm for principal coordinate analysis (PCoA). We tested the samples in the three groups using the Kruskal–Wallis test based on the PC1 and PC2 principal components. The results revealed statistically significant differences among the three groups (Figure 3).

#### 3.1.3. Microbial Community Structure

**Figure 4:**
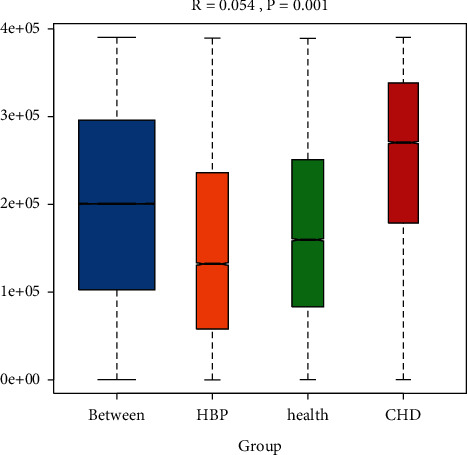
Analysis of similarities (ANOSIM) among the three groups (the range of *R* is [−1, 1]. *R* > 0 indicates that the difference between groups is greater than that within groups. *P* value can be obtained by the permutation test).

According to the analysis of similarities (ANOSIM) (Figure 4), We can see that *R* = 0.054 and *P*=0.001, indicating that the difference between groups is significantly greater than that within groups.

**Figure 5:**
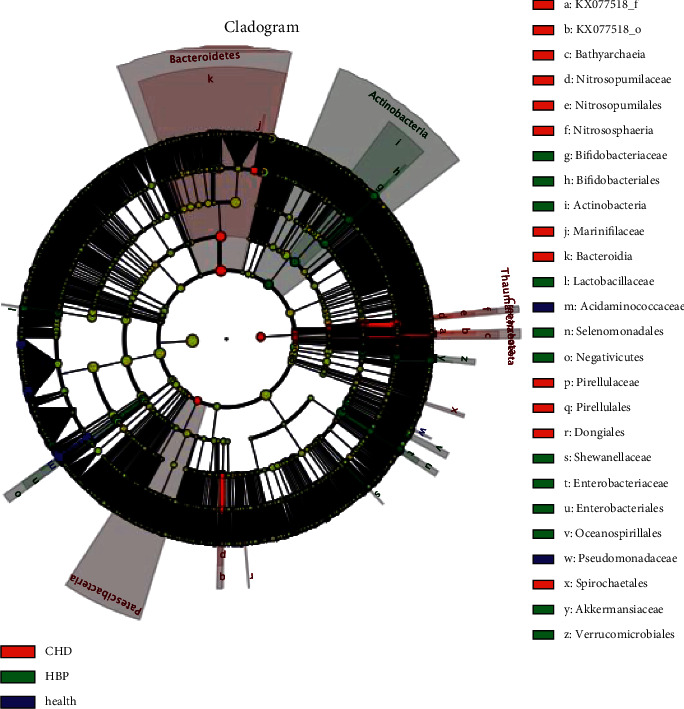
Cladogram shows the most differentially abundant taxa.

**Figure 6:**
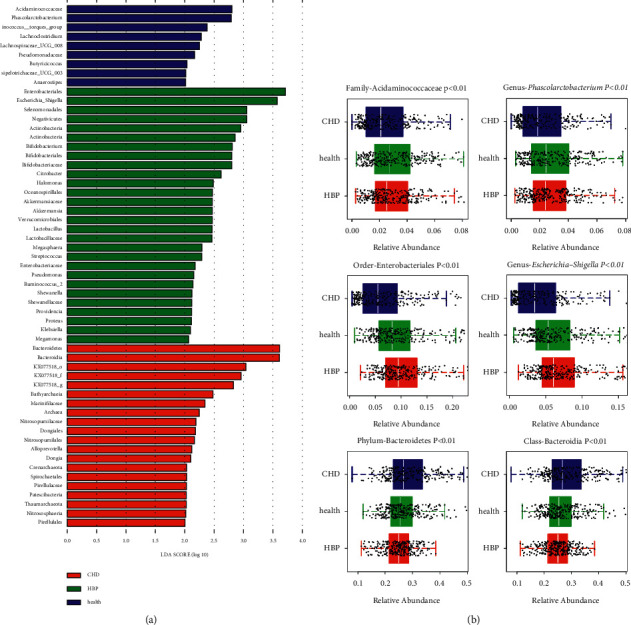
LEfSe analysis with LAD score >2.

In Figure 5, we can see five circles of different sizes from the inside to the outside, representing five different classifications of phylum, class, order, family, and genus. In Figure 6, we can see the CHD group, Bacteroidetes and Bacteroidia were the dominant bacteria; in the HBP group, Enterobacteriales and *Escherichia-shigella* were predominant, and in the control group, Acidaminococcaceae and *Phascolarctobacterium* were the dominant bacteria.

#### 3.1.4. Receiver Operating Characteristic (ROC) Curve

**Figure 7:**
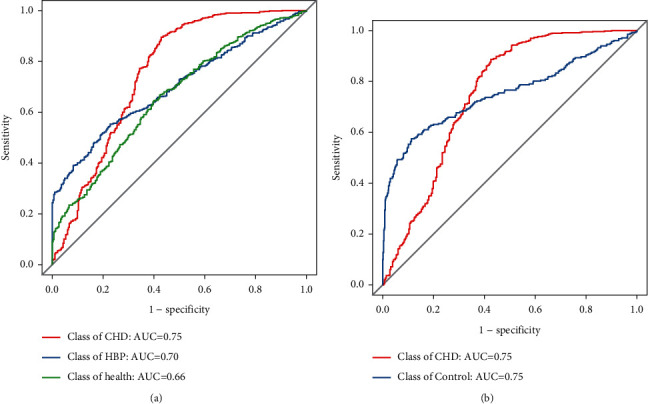
ROC curve among the control, HBP, and CHD groups.

At the genus level, the results indicated that the value of the area under the curve (AUC) in the three groups was 0.75, 0.70, and 0.66, respectively (Figure 7(a)). Additionally, we combined patients with HBP with healthy controls to form a control group for differential detection against patients with CHD. The results showed that the AUC of the new model reached up to 0.75 (Figure 7(b)).

#### 3.1.5. Flora Metabolic Function Prediction

We applied the BugBase to predict the phenotypes of gut microbiota and conducted the intergroup statistical test. As a result, Aerobic, Anaerobic, Contains_Mobile_Elements, Facultatively_Anaerobic, Forms_Biofilms, and Stress_Tolerant were significantly different (*P* < 0.05), but we observed no significant difference in Gram-positive bacteria, Gram-negative bacteria, and pathogenicity (*P* > 0.05) (Table 3) (Attachment 1 (available here)).

### 3.2. Discussion

In recent years, the results of many clinical and animal studies have shown that microbiota disorders are closely related to the occurrence of diseases [18–25]. Previous studies by Chan et al. [26] showed that the changes of bacterial genus and the decrease of *α*-diversity were significantly correlated with the size of atherosclerotic plaque (*P* < 0.05). Other studies have also shown that the increase of *Enterobacteriaceae* is associated with the appearance of atherosclerotic plaque size and more severe coronary atherosclerosis (*P* < 0.05) [27, 28]. Our findings are consistent with the results of a study with a sample size of forty people by Gózd-Barszczewska et al. [29] suggesting that *Bacteroides* may play a role in patients with coronary heart disease. The results of our study suggest that there is no difference in microbiota diversity between patients with hypertension and patients with coronary heart disease (*P* > 0.05), which is inconsistent with Liu's study [30]. The inconsistency may be caused by the regional concentration or ethnic correlation of samples. The study by Cui et al. [31] showed that the significant decrease in the proportion of Bacteroidetes in the CHD group might be negatively correlated with the risk of coronary heart disease (*P* < 0.05). However, our study showed that the quantity of Bacteroidetes on the genus level in the CHD group was higher than that of the healthy control group, which may be because our study considers quantitative aspects. Finally, we expected more than 90% accuracy in ROC curve predictions, but achievements were not ideal, reaching only 75%. This discrepancy may be related to the age, weight, and dietary habits of the population [32, 33].

Our study was the first to use a large amount of clinical sample data. Such a large sample database can improve the accuracy of data analysis and model accuracy, and Chen et al. [34] and Brown and Hazen [35] suggested that differences in gut microbiota can be used to predict cardiovascular disease. The ultimate goal is to optimize and develop the whole process from sampling device to extraction to library building and sequencing analysis and to achieve rapid, low-cost detection of intestinal flora in a large population [36].

Our study also had several limitations. First, all the selected population came from the First Affiliated Hospital of Zhejiang Chinese Medicine University, which had obvious geographical limitations. Second, the inclusion and exclusion criteria of this study were strictly controlled, resulting in limited participation. Third, the heterogeneity of clinical samples leads to the decrease of repeatability of experimental result. Finally, to increase the sample size, the age span should be larger.

The implication of our study is that the microbiome may serve as a biomarker to predict cardiovascular disease, but this must be further revealed by the characterization or enrichment of the specific bacterial community that the microbiome diagnoses [37]. At the same time, the relationship between intestinal microbiota and different CHD subtypes and different cardiovascular risk levels of HBP needs to be further explored.

## Figures and Tables

**Table 1:** Demographic and clinical data of patients and healthy controls (the chi-squared test (sex), one-way ANOVA (age, BMI)).

	Control	HBP	CHD	F	*P* value
Number	300	300	300	—	—
Sex (M/F)	165/135	143/157	154/146	—	0.199
Age (Y)	62.02 ± 11.79	61.60 ± 11.92	60.53 ± 10.48	1.367	0.255
BMI (kg/m^2^)	20.64 ± 1.85	20.47 ± 2.01	20.54 ± 1.95	0.626	0.535

**Table 2:** Statistics of sequences.

Group	Number_sample	Sum_tags	Average_tags	Max/Min	OTUs
Control	300	12403888	41346	60761/28043	628
HBP	300	12797717	42659	60581/28001	647
CHD	300	25240725	84135	149768/28645	775

**Table 3:** Flora metabolic function prediction among the three groups.

BugBase	KS_*P* value
Aerobic	0.0161
Anaerobic	<0.01
Contains_Mobile_Elements	<0.01
Facultatively_Anaerobic	<0.01
Forms_Biofilms	<0.01
Gram_Negative	0.6225
Gram_Positive	0.6225
Potentially_Pathogenic	0.3747
Stress_Tolerant	<0.01

## Data Availability

The datasets used and/or analyzed during the current study are available from the corresponding author.

## Supplementary Materials

Supplementary Materials Class.Groups.sig.boxplot.pdf. Family.Groups.sig.boxplot.pdf. Genus.Groups.sig.boxplot.pdf. Order. Groups.sig.boxplot.pdf. Phylum.Groups.sig.boxplot.pdf. Attachment 1: flora metabolic function prediction.
