# Supplementary figures and images for: Analysis of Gut Microbiota in Patients with Coronary Artery Disease and Hypertension
Source: Evid Based Complement Alternat Med. 2021 Dec 27;2021:7195082. doi: 10.1155/2021/7195082 (PMC8723847; doi:10.1155/2021/7195082)

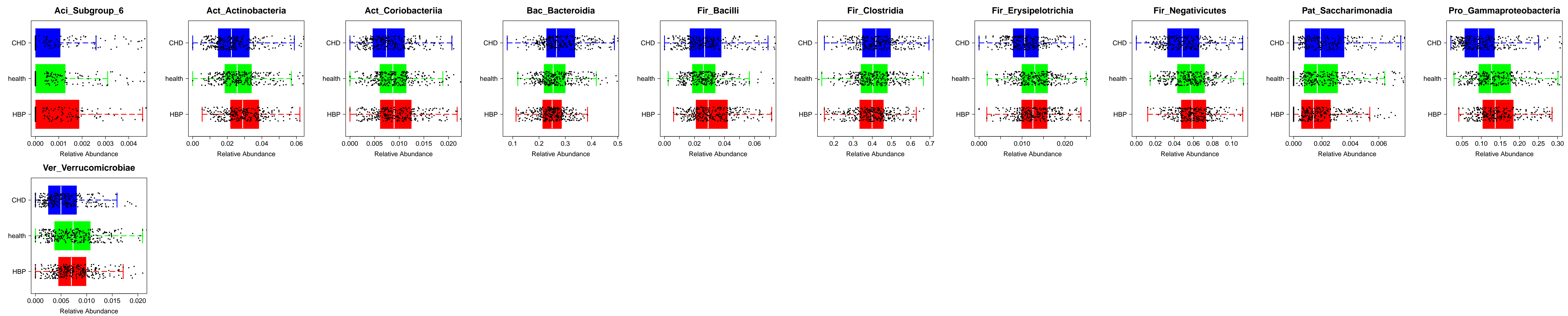

Supplement: Supplementary Materials — Class.Groups.sig.boxplot.pdf. Family.Groups.sig.boxplot.pdf. Genus.Groups.sig.boxplot.pdf. Order. Groups.sig.boxplot.pdf. Phylum.Groups.sig.boxplot.pdf. Attachment 1: flora metabolic function prediction. [file 7195082.f1.zip › 7195082.f1/Class.Groups.sig.boxplot.pdf]

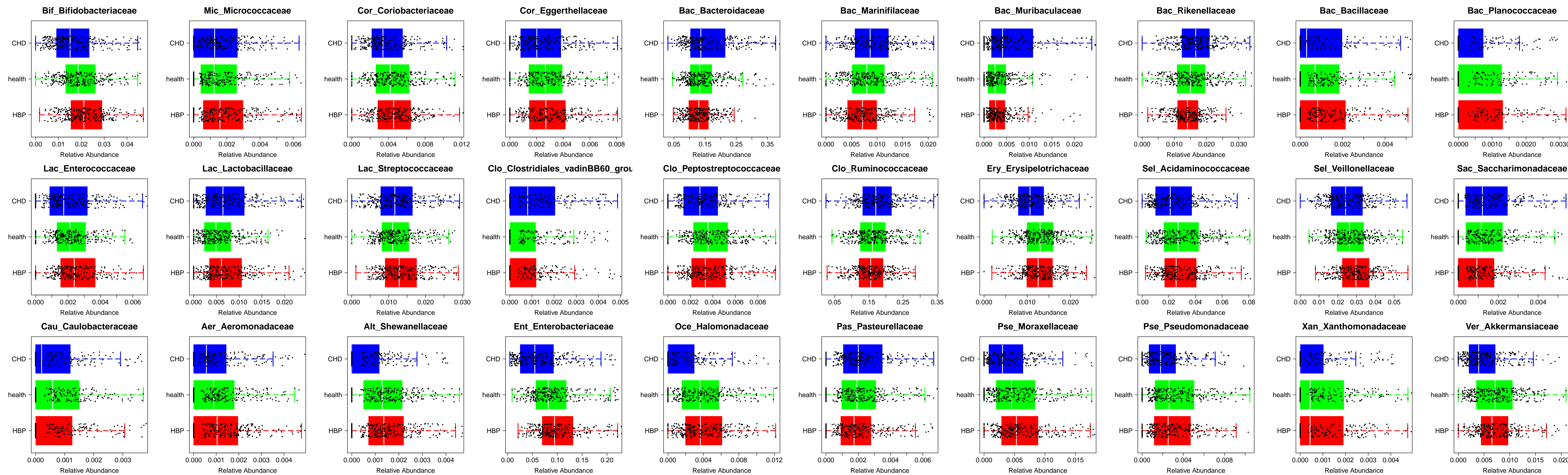

Supplement: Supplementary Materials — Class.Groups.sig.boxplot.pdf. Family.Groups.sig.boxplot.pdf. Genus.Groups.sig.boxplot.pdf. Order. Groups.sig.boxplot.pdf. Phylum.Groups.sig.boxplot.pdf. Attachment 1: flora metabolic function prediction. [file 7195082.f1.zip › 7195082.f1/Family.Groups.sig.boxplot.pdf]

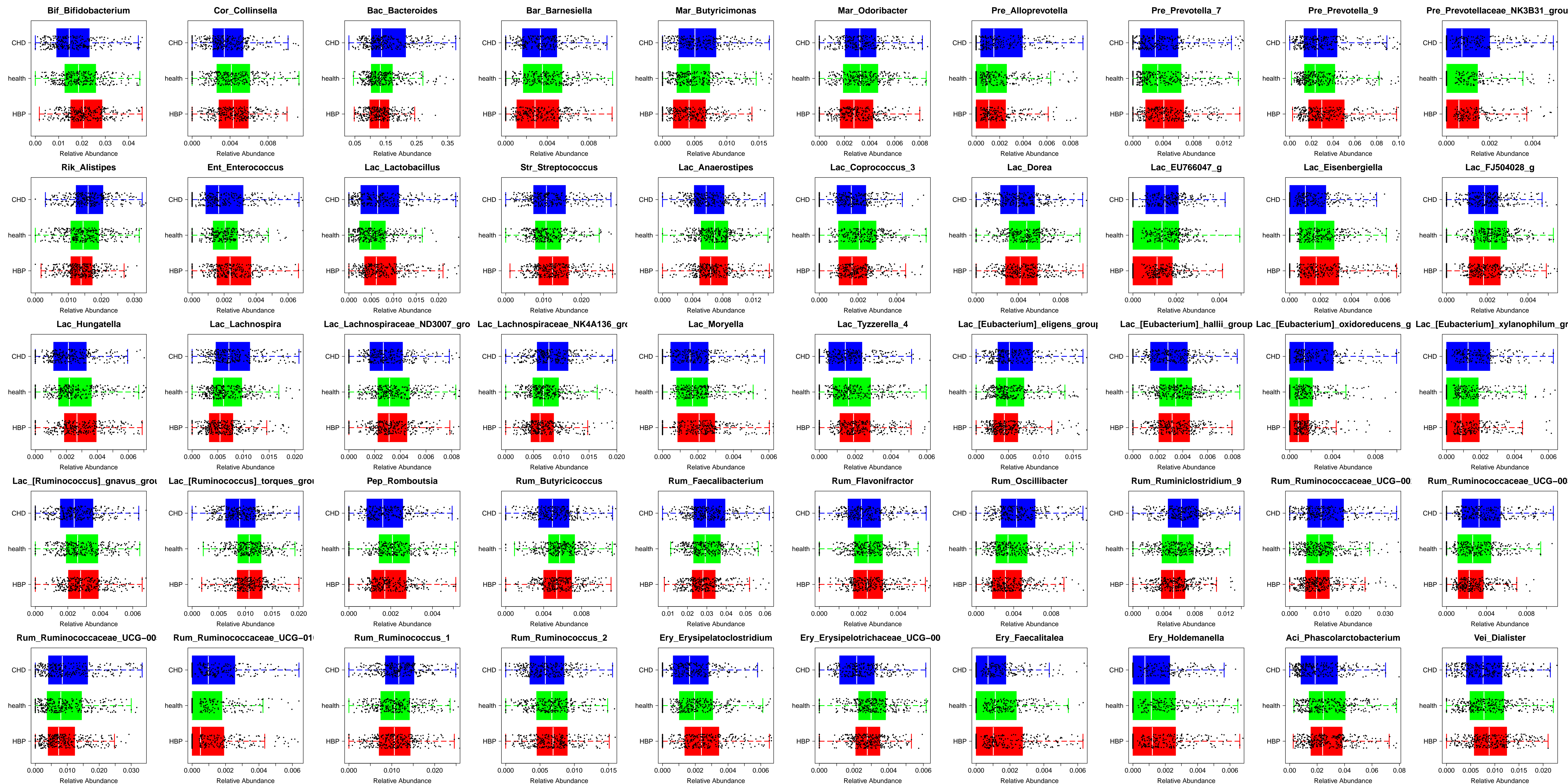

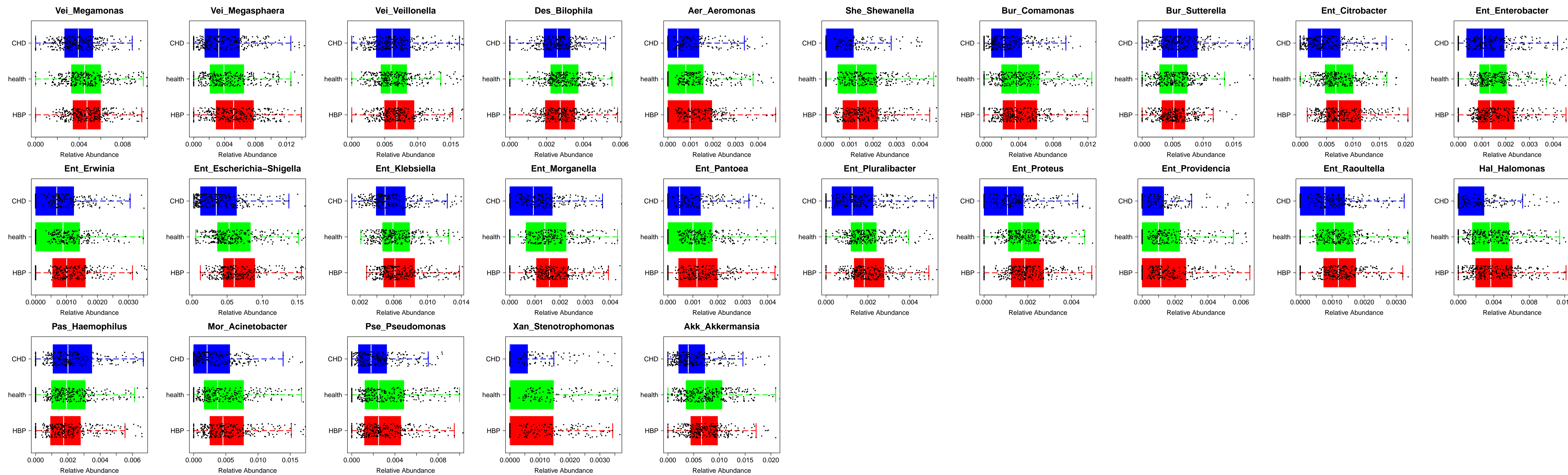

Supplement: Supplementary Materials — Class.Groups.sig.boxplot.pdf. Family.Groups.sig.boxplot.pdf. Genus.Groups.sig.boxplot.pdf. Order. Groups.sig.boxplot.pdf. Phylum.Groups.sig.boxplot.pdf. Attachment 1: flora metabolic function prediction. [file 7195082.f1.zip › 7195082.f1/Genus.Groups.sig.boxplot.pdf]

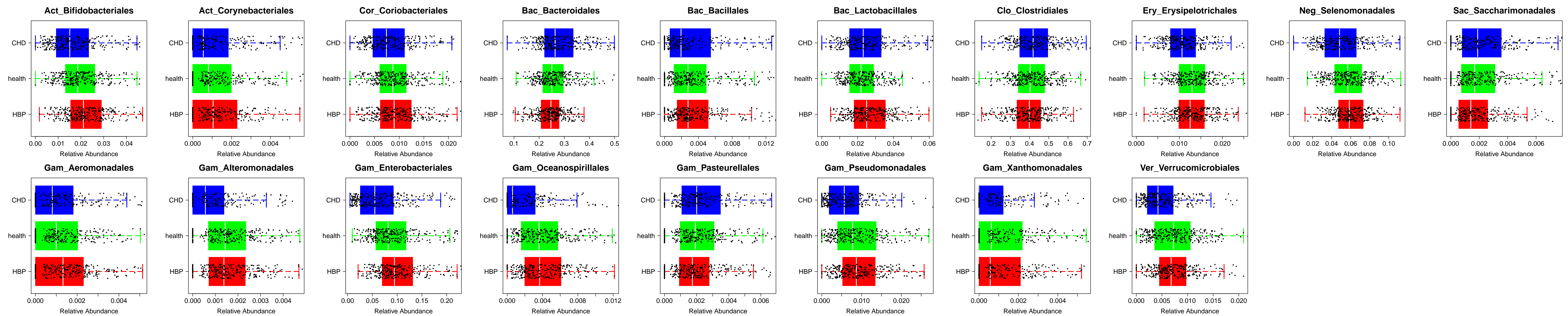

Supplement: Supplementary Materials — Class.Groups.sig.boxplot.pdf. Family.Groups.sig.boxplot.pdf. Genus.Groups.sig.boxplot.pdf. Order. Groups.sig.boxplot.pdf. Phylum.Groups.sig.boxplot.pdf. Attachment 1: flora metabolic function prediction. [file 7195082.f1.zip › 7195082.f1/Order.Groups.sig.boxplot.pdf]

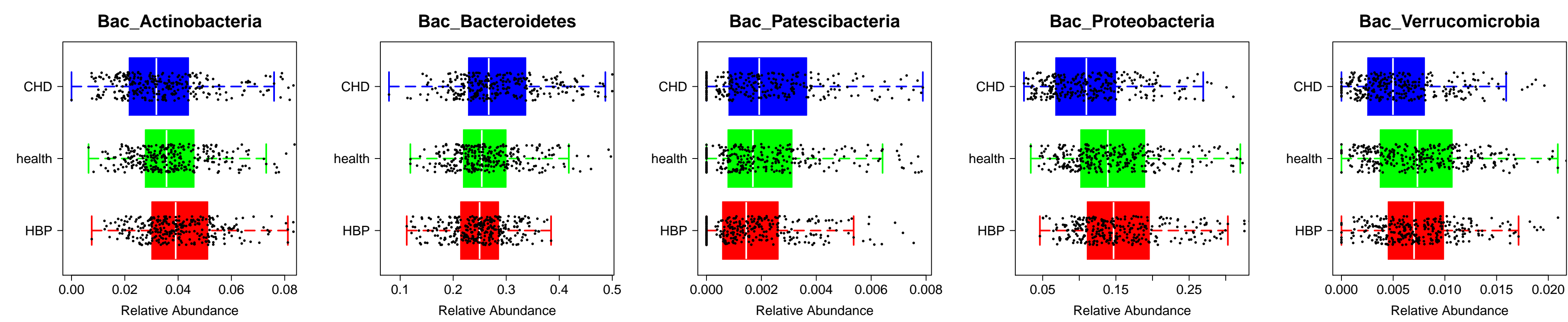

Supplement: Supplementary Materials — Class.Groups.sig.boxplot.pdf. Family.Groups.sig.boxplot.pdf. Genus.Groups.sig.boxplot.pdf. Order. Groups.sig.boxplot.pdf. Phylum.Groups.sig.boxplot.pdf. Attachment 1: flora metabolic function prediction. [file 7195082.f1.zip › 7195082.f1/Phylum.Groups.sig.boxplot.pdf]
