# Supplementary material for: Analysis of Gut Microbiota in Patients with Coronary Artery Disease and Hypertension
Source: Evid Based Complement Alternat Med. 2021 Dec 27;2021:7195082. doi: 10.1155/2021/7195082 (PMC8723847; doi:10.1155/2021/7195082)
Supplement: Supplementary Materials — Class.Groups.sig.boxplot.pdf. Family.Groups.sig.boxplot.pdf. Genus.Groups.sig.boxplot.pdf. Order. Groups.sig.boxplot.pdf. Phylum.Groups.sig.boxplot.pdf. Attachment 1: flora metabolic function prediction. [file 7195082.f1.zip › 7195082.f1/Attachment 1.Flora metabolic function prediction .doc]

Aerobic：

Anaerobic：

Contains_Mobile_Elements

Facultatively_Anaerobic

Forms_Biofilms

Gram_Negative

Gram_Positive

Potentially_Pathogenic

Stress_Tolerant
